# Miniaturized Sensors for Detection of Ethanol in Water Based on Electrical Impedance Spectroscopy and Resonant Perturbation Method—A Comparative Study

**DOI:** 10.3390/s22072742

**Published:** 2022-04-02

**Authors:** Angelo Leo, Anna Grazia Monteduro, Silvia Rizzato, Angelo Milone, Giuseppe Maruccio

**Affiliations:** Omnics Research Group, Department of Mathematics and Physics, University of Salento, CNR-Institute of Nanotechnology, INFN Sezione di Lecce, Via per Monteroni, 73100 Lecce, Italy; silvia.rizzato@unisalento.it (S.R.); angelo.milone@unisalento.it (A.M.); giuseppe.maruccio@unisalento.it (G.M.)

**Keywords:** dielectric sensors, electrical impedance spectroscopy, split ring resonators, resonant perturbation methods, equivalent circuit analysis, ethanol

## Abstract

The development of highly sensitive, portable and low-cost sensors for the evaluation of ethanol content in liquid is particularly important in several monitoring processes, from the food industry to the pharmaceutical industry. In this respect, we report the optimization of two sensing approaches based on electrical impedance spectroscopy (EIS) and complementary double split ring resonators (CDSRRs) for the detection of ethanol in water. Miniaturized EIS sensors were realized with interdigitated electrodes, and the ethanol sensing was carried out in liquid solutions without any functionalization of the electrodes. Impedance fitting analysis, with an equivalent circuit over a frequency range from 100 Hz to 1 MHz, was performed to estimate the electric parameters, which allowed us to evaluate the amount of ethanol in water solutions. On the other hand, complementary double split ring resonators (CDSRRs) were optimized by adjusting the device geometry to achieve higher quality factors while operating at a low fundamental frequency despite the small size (useful for compact electronic packaging). Both sensors were found to be efficient for the detection of low amounts of ethanol in water, even in the presence of salts. In particular, EIS sensors proved to be effective in performing a broadband evaluation of ethanol concentration and are convenient when low cost is the priority. On the other end, the employment of split ring resonators allowed us to achieve a very low limit of detection of 0.2 *v*/*v*%, and provides specific advantages in the case of known environments where they can enable fast real-time single-frequency measurements.

## 1. Introduction

Portable and low-cost sensors for the detection of chemicals in liquid are finding an ever growing demand in many application fields, such as in the food industry [1,2], medical diagnostics [3,4,5,6] and environmental monitoring of contaminants and pollutants [7].

In this contest, the development of sensors for the evaluation of ethanol content in liquid is particularly important for monitoring processes in distilleries and breweries and in the manufacture of drugs, plastics, lacquers, polishes, plasticizers and cosmetics [8]. Indeed, it is largely used for its miscibility in water and as a solvent in several processes. It is also used as an antiseptic, thanks to its ability to kill microorganisms by denaturing their proteins and dissolving fats. However, the main consumer is the automotive sector: approximately 73% of the ethanol produced in the world is destined to be an additive for fuels, in blends with variable percentages; moreover, bioethanol (along with biodiesel) is now considered one of the most promising alternatives to fossil fuels and is the main biofuel used as a substitute for road transport vehicles.

The evaluation of the concentration of ethanol in aqueous solutions makes use of a variety of techniques [9], among which gas chromatography stands out for alcoholic beverages and clinical samples. Alternative methods for the detection of ethanol in liquids are based on indirect calculations of ethanol content or direct determination by oscillating density meters [10] and UV-Vis-IR spectroscopy [11,12,13], respectively. Through the first method, the determination of ethanol is carried out by a three-step protocol, which consists of flow injection, pervaporation and density measurements in mixtures, making this method accurate but time-consuming. In addition, infrared spectroscopy involves the use of a spectrophotometer and is accurate but restricted to lab settings. On the other side, cheaper, portable devices, such as hydrometers controlled by an artificial neural network [14], have poor resolution and a strong temperature dependency, which makes them unsuitable for accurate monitoring of ethanol content over the typical time of the fermentation process.

Other methods for ethanol detection are based on the employment of sensitive hydrogels, whose swelling–shrinking state after contact with ethanol can be interrogated, for example, by pressure sensors, quartz crystal microbalances or optically, leading to discernible ethanol concentration variations of 10% [15]. Further state-of-art ethanol sensors are based on multilayer integrated optical waveguides [16] and electrochemical responsive microring resonators, nanodots and nanowires [17,18,19]. They show excellent sensitivity, but their use is strongly connected to environmental conditions: optical sensors require instrumentation that is difficult to implement in small devices, moreover the electrochemical ones often suffer from high temperatures. RF sensors are more suitable for the industrial field with respect to the aforementioned ones since they provide the benefits of being low cost, contactless and reusable, while maintaining excellent performances.

In this respect, electrical impedance spectroscopy (EIS)-based sensors [20] and split-ring resonators metamaterial structures provide interesting alternatives, which have already proven useful for the evaluation of ethanol concentration [21,22,23,24] and are suitable for integration in microfluidic platforms [25,26,27,28]. However, few works have reported miniaturized EIS sensors with interdigitated (ID) electrodes and without any functionalization for ethanol sensing in liquid solutions. Furthermore, in these studies, only the capacitance values at a fixed frequency have been typically taken into account to sense the amount of ethanol in a solution [29,30], and none of them performed complex impedance fitting analysis with an elemental equivalent circuit. Here we employed this different approach, showing how the estimated electric parameters allow the evaluation of the ethanol amount in water solutions, providing additional insight. Furthermore, we also employed complementary double split ring resonators (CDSRRs) as compact two-port sensors with optimal field confinement for ethanol content evaluation by resonant perturbation methods. Thanks to an optimized device geometry, we achieved higher quality factors with respect to literature values while operating at a low fundamental frequency, despite the small size (useful for a compact electronic packaging). The performances of these two sensing approaches have been compared for their ability to detect ethanol presence in water.

## 2. Materials and Methods

### 2.1. Fabrication of Electrical Impedance Spectroscopy (EIS) Devices and Experimental Setup

**Figure 1:**
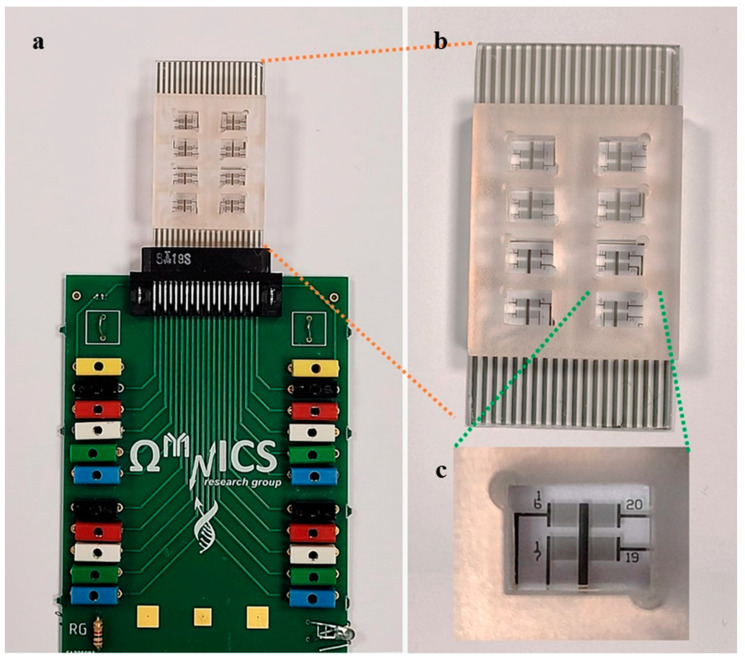
(**a**) EIS sensor with IDT electrodes and 3D resin chamber connected to the printed circuit board; (**b**) details of the EIS sensor device in which the electrodes for external connections are visible on the bottom and on the top of the device; and (**c**) enlargement of a sensor area in which a common reference and four signal pins are clearly visible.

The electrical impedance spectroscopy (EIS) device employed for ethanol concentration evaluation was composed of a sensing module with interdigitated electrodes and an array of resin 3D printed chambers, which separated each sensing area (Figure 1). A similar layout was previously used for biosensors and environmental sensor applications [31,32,33,34]. In detail, each sensing module consisted of four arrays, each one containing four couples of interdigitated microelectrodes as transducers, which had a common reference and four signal pins (Figure 1c). These electrodes, characterized by a finger width and interfinger gap of 10 μm, were fabricated by photolithography and lift-off process on glass substrates (EOT) using a Karl Suss MA6 mask aligner, AZ5214B resist and thermal evaporation of Cr/Au (3 nm/35 nm). The array of polymeric chambers (50 μL volume for each one) was first designed using AUTOCAD and then realized using an Asiga Max 3D printer. The impedance measurements were carried out at room temperature employing an Agilent E4980A precision LCR meter and homemade software written in Labview (GM-Multiscan) for the data acquisition. An AC-driving voltage of 100 mV was applied over a frequency range from 100 Hz to 1 MHz. The electrical connections between the EIS chip and the LCR meter were performed, fabricating dedicated pads on the EIS chip with a specific pitch to match a commercial connector integrated on a printed circuit board (PCB) (Figure 1a,b).

### 2.2. Design, Simulation and Fabrication of CDSRR-Based Devices and Experimental Setup

**Figure 2:**
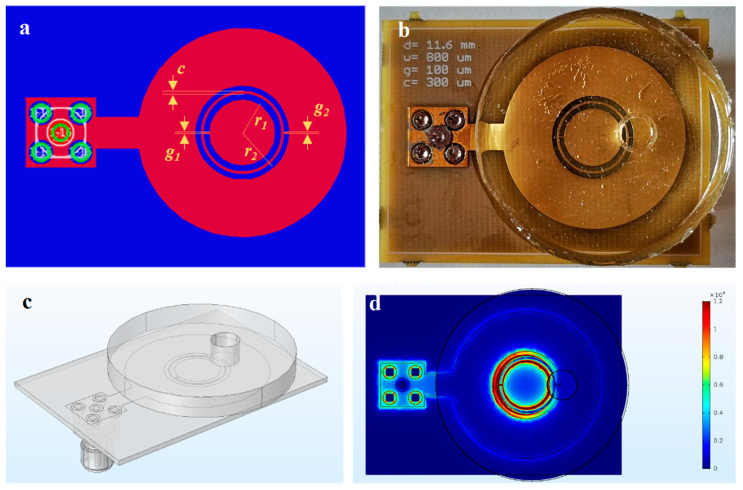
(**a**) CAD project of CDSRR. The upper metallized region is indicated in red, while the ground is represented in blue; vias are shown as green circular crowns. Particular features are indicated in yellow. (**b**) Device covered by a PDMS disk in which the well was realized. (**c**) 3D CAD drawn with Comsol© Multiphysics 5.3a for the whole device. (**d**) Module of the RF electric field in first mode for a slice adjacent to the interface between the resonator and the PDMS disk.

The device exploited for the detection of ethanol concentration in water by resonant perturbation methods was a one-port complementary double split ring resonator (CDSRR), realized in a Cu disk of 27 mm diameter and 15 µm height, connected to a right-angle SMA through a metallized plate (the feedline) of dimensions 4 mm × 6 mm, as shown in Figure 2a. Peculiar attention was paid to the line width design for lossy coupling the devices to instruments and for promoting resonance. The lossy coupling regime could be obtained by acting on the impedance of entire devices at analytically estimated fundamental modes. In particular, a good performance could be ensured by modeling (i) the feed line to have matched impedance (Z ≃ 50 Ohm) near connectors and high impedance (~100 Ohm) near resonators, and (ii) the ring to have impedance in the range 50 Ω < Z < 100 Ω. By employing KiCad software, we extracted the circuit length and line width by setting the impedance conditions and losses in both the conductor and substrate. 

The resonator was composed of two concentric rings with mean radii r_1_ and r_2_ of 4.7 mm and 5.8 mm, respectively, width of 0.8 mm and separated by a coupling gap c of 0.3 mm. Each ring showed a 100 µm slit (g_1_ = g_2_), which lay on the axis joining the center of the resonator to the signal via.

The built-up board was constituted by top and bottom copper layers of 12–18 µm, and a substrate of improved-FR4 (IS400), which was 1 mm thicker and with permittivity ε_r_ ranging from 4.43 to 4.51 for frequencies among 500 MHz to 20 GHz and loss tangents δ_t_ of 0.0149 ÷ 0.0189 in the same spectrum.

For the ethanol sensing application, the resonator was then covered by a polydimethylsiloxane (PDMS) disk of 4.2 mm height and 35 mm diameter, in which a hole of 5.5 mm in diameter was made in order to obtain access to the sensing area. The PDMS disk was attached to the device by applying a thin layer of liquid PDMS and placing the system in the oven for 30 min at 70 °C. In such a way, a well with a total capacity of 100 µL was realized. The whole CDSRR device is shown in Figure 2b.

The sensing experiments were carried out directly by connecting the device to a Keysight E5061B ENA Vector Network Analyzer by means of SMA connectors to measure the module of the scattering parameter S_11_, thus totally eliminating distortion effects due to the presence of cables. In these conditions, the resonator showed the first resonance peak at 1.094 GHz, with an amplitude of −16.37 dBm, indicating a quality factor Q ~ 100 (for the empty sensor).

To evaluate how the electric field was distributed in proximity of CDSRR and to test the performances of the projected ring before the fabrication, Comsol© Multiphysics 5.3a (RF module) was employed to simulate the reflection spectrum. Therefore, we first designed the CAD of the whole device as reported in Figure 2c, including the PDMS disk with the well, the SMA connector and a big sphere to take into account the environment (not shown for clarity). For each material, we inserted the values of electrical conductivity, dielectric constant and magnetic permeability taken from the literature. We found that the first mode, which was experimentally used, was characterized by an eigenfrequency of 1.081 GHz, with a relative discrepancy with respect to measurements less than 2%; the mismatch could be further reduced by using real values of the electrical characteristics. From the simulation on the first mode, it was possible to notice the RF electric field distribution at the interface between the PDMS disk and the ring itself. As reported in Figure 2d, the presence of the well in proximity to the external slit deeply modified the field distribution, which resulted in it not being symmetric with respect to the center of the ring. Therefore, the presence of a solution inside the well significantly perturbed the resonator, as the dielectric constant of material increased.

## 3. Results and Discussion

### 3.1. Detection of Ethanol Using the EIS Sensor

**Figure 3:**
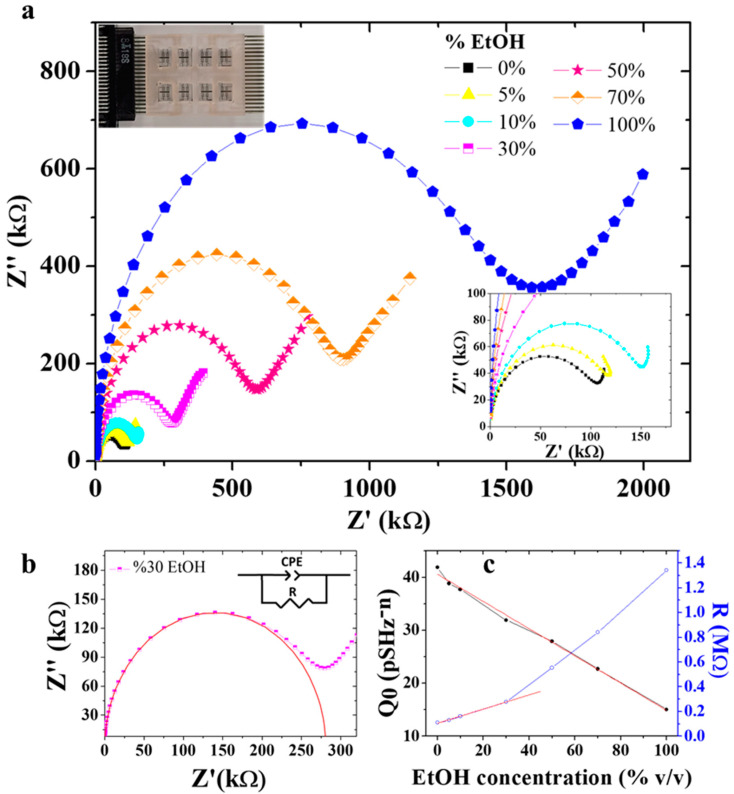
(**a**) Nyquist plots of EIS sensors by varying the %EtOH in DI water. Insets: (top left) optical image of the EIS sensor connected to the PCB for impedance measurements; (bottom down) Details of the impedance responses for pure water (black squares) and with a low concentration of ethanol, 5% (yellow triangles) and 10% (cyan dots). (**b**) Fitting of Nyquist plot recorded for the solution with 30% EtOH (inset: equivalent circuit employed to model the impedance responses acquired for different ethanol contents). (**c**) Q_0_ (black full dots) and R (blue empty dots) profiles as a function of ethanol concentration.

Dielectric impedance spectroscopy is a powerful technique for material characterization and sensing [35,36,37,38,39]. In Figure 3a, the impedance response of a typical EIS sensor is reported by varying the concentration of ethanol in demineralized (DI) water from 0% to 100%. During the experimental measurements, a resin chamber over a sensing area was filled with 50 µL of sample solution and the impedance data were acquired over a frequency range from 100 Hz to 1 MHz. The solution was then removed and the well was washed with DI water and dried with nitrogen before recording the impedance response of a solution with a different ethanol percentage content. The impedance data were presented as Nyquist plots, in which the imaginary part of impedance (Z″) is plotted as a function of the real part (Z′). Figure 3a shows that increasing the amount of ethanol in the solution results in an increase in the arc diameter, reaching the maximum value with absolute ethanol (blue pentagons in the graph) and the minimum with DI water (black squares in the graph). In order to promote a more in depth understanding on how the electric parameters changed by varying the ethanol amount in the water solution, the complex impedance data (Z′, Z″) were analyzed with the elemental equivalent circuit method. First we selected the most suitable equivalent circuit analyzing the complex impedance of water, and then we employed the same equivalent circuit to fit the impedance data recorded by varying the ethanol concentration in water.

The equivalent circuit, which better described our data, is reported as inset in Figure 3b. It consists of a parallel between a constant phase element (CPE) and a resistance (R). This is the most common circuit used to describe a not-ideal capacitor, characterized by a distribution of relaxation times [35,36,38]. The impedance of the CPE element, Z_CPE_, is expressed as follows:

(1) $$Z_{\mathrm{CPE}}=\frac{1}{Q_{0}{\left(j\omega \right)}^{n}}$$

where Q_0_ is expressed in SHz^−n^, ω = 2πf is the angular frequency and 0 < n < 1, which corresponds to an ideal capacitor when n = 1 and to an ideal resistor when n = 0, j is the imaginary unit.

To give an idea on how the selected circuit describes our experimental data, we report in Figure 3b the fit (red line) of the impedance data acquired for a 30% amount of EtOH in solution by using the CPE-R equivalent circuit. The electrical parameters, Q_0_ and R, extracted by fitting the Nyquist plots are reported in Figure 3c as a function of the % EtOH in the solution. An almost linear trend is observed for Q_0_ (black full dots) on the entire EtOH percentage range investigated, while the resistance, R (blue empty dots), shows a variation of the slope for EtOH concentration lower than 30%. Moreover, since Q_0_ is related to the capacitive properties of the liquid phase, the data obtained suggest a reduction of the dielectric permittivity of the solution by increasing the EtOH content. This is in agreement with the dielectric properties of the pure liquid, since a dielectric constant of about 80 is known in the literature for DI water [40,41] and a permittivity of about 25 for absolute EtOH [42,43,44]. On the other hand, the increase in the resistance by increasing the ethanol content in the solution agrees with the lower conductivity of ethanol, with respect to the water one.

We evaluated the limit of detection (LOD) of the sensor by dividing the Q_0_ and R uncertainties (calculated considering the average of residuals from the linear fits) with the sensitivity, represented by the slope of the linear fits of the two parameters considered. Specifically, we linearly fitted Q_0_ over the whole percentage variation of ethanol and found a sensitivity of 0.257 ± 0.008 pSHz^−n^ for 1% increments of EtOH content in water and a LOD of 2.5% EtOH, while the resistance variation was linearly fitted from 0% to 30% EtOH, estimating a sensitivity of 5.74 ± 0.26 kΩ/% EtOH and a LOD of 0.7% EtOH.

### 3.2. Detection of Ethanol Using the CDSRR Sensor

Resonance perturbation methods were also employed for material characterization and sensing purposes [45,46,47]. The CDSRR sensor was tested for the detection of EtOH in the liquid phase by depositing in the PDMS well a 100 µL droplet of ethanol-DI water solution. The concentration of ethanol in water was varied from 0% to 100%. As for EIS sensors, after each measurement, the solution was removed, the well was washed with demineralized water and then dried with nitrogen flow, bringing the system to its initial conditions, with a maximum frequency shift and a maximum amplitude variation of less than 0.2%.

**Figure 4:**
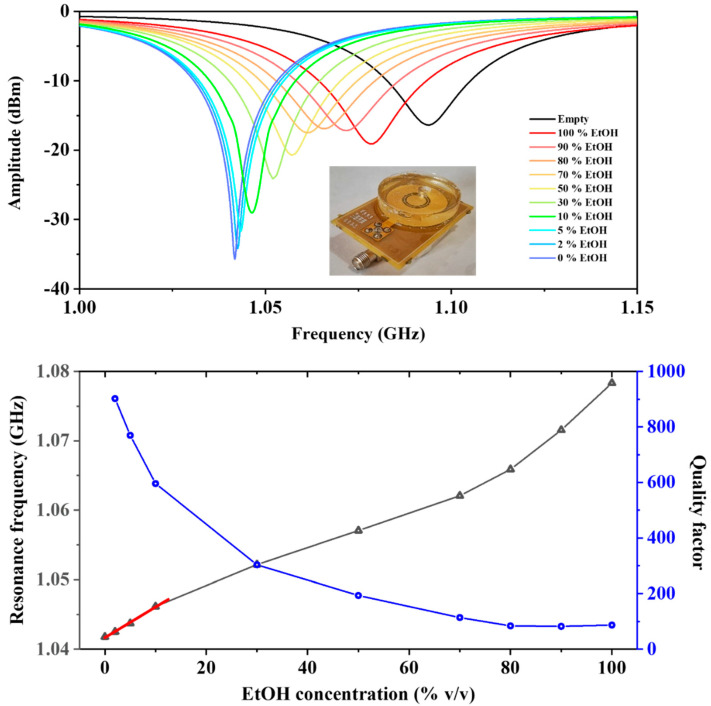
(**top**) Measured S_11_ curves for various water-ethanol mixtures at different volume fractions. Inset: optical image of the CSRR sensor. (**bottom**) Resonant frequency and quality factor for different water-ethanol volume fractions. Linearization of the response for the first four investigated concentrations is reported through a red line.

The reflection signal module S_11_ amplitudes from loaded devices are reported in Figure 4 (top panel). Device loading by ethanol/water solution caused a frequency shift and an amplitude variation of the resonant peak in addition to a modification of quality factor Q of the resonator. In Figure 4 (bottom panel), the variation of the resonance frequency and Q values as a function of the ethanol concentration are shown. By reducing the concentration of EtOH, an almost linear shift of the resonance peak toward lower frequencies was observed, reaching its minimum with water (1.042 GHz).

On the other hand, an increase in the resonator quality factor Q was observed, passing from about 100 for absolute EtOH (as for the empty device value) to about 1000 for pure water. By fitting the resonance frequencies of the loaded device with respect to the concentration of ethanol in water up to 10% (red line in Figure 4 (bottom panel)), we obtained a sensitivity of 0.329 ± 0.016 MHz for 1% increments of EtOH content in water. Estimation of LOD of CSRR was then carried out by dividing the resonance frequency uncertainty with the sensitivity, obtaining a LOD of 0.2% of EtOH.

The high sensing performance of our CDSRR sensors was mainly associated to the improved quality factor of the unloaded device, which, in our case, reached values higher (at least double) than the ring resonators exploited in the present literature for the evaluation of ethanol content in water [25,48,49,50,51]. Moreover, the relevant variation of quality factor observed in our CDSRRs (about 18 times from water to pure ethanol) could be also exploited for evaluation of dielectric losses of the investigated solutions.

### 3.3. Discussion

In Table 1, the values of sensitivity and LOD for both EIS and CDSRR devices for ethanol content detection in DI water are summarized and compared with the performance of devices based on similar sensing principles. In particular, the two rows related to our EIS sensors show both values of sensitivity and LOD obtained by the two electric parameters Q_0_ and R trends, respectively, while the last row refers to the sensitivity and LOD of loaded CDSRR established from the evaluation of resonance frequency f_res_ variation. It was evident that the better LOD was obtained using CDSRR, i.e., resonance perturbation methods. Notably the only report with better LOD [28] needed a transmission-matrix formalism analysis.

Since the response and performance of the sensors could also be expected to be influenced by the presence of other compounds in the solutions, further experiments were carried out with both EIS and CDSRR sensors to investigate and provide information on the sensors response in a different, more complicated environment, such as in the presence of water-soluble contents (e.g., ions, salts), an aspect which could be relevant for applications. In particular, we employed a normal saline (NS) solution—NaCl 0.9% *w*/*v*—instead of DI water to assess the influence of the presence of ions as a case of study.

**Figure 5:**
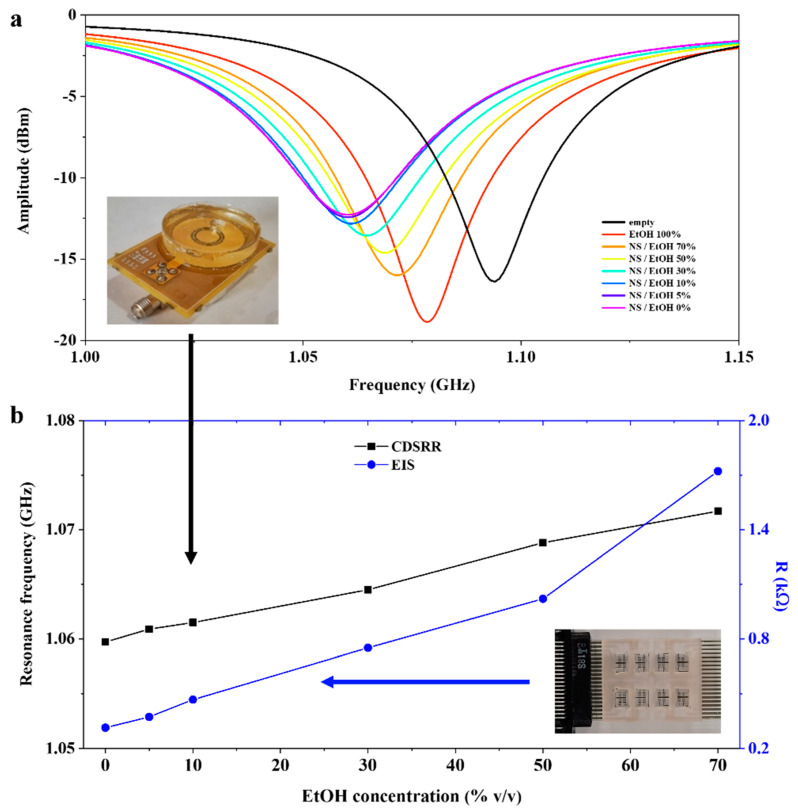
(**a**) Measured S_11_ of CDSRR curves for various NS-ethanol mixtures at different volume fractions. (**b**) Resistance (R) and resonant frequency for different NS-ethanol volume fractions extracted with EIS and CDSRR sensors, respectively.

As reported in Figure 5, both sensors were able to identify the ethanol presence also in these conditions. However, the increase in solution conductivity affected their responses. Concerning CDSRR, as shown in Figure 5a, the effect of the NS solution was evident both on the shift of the resonant frequency of the loaded CDSRR and on the reduction in the quality factor. The CDSRR loaded with pure NS solution showed a resonance frequency of 1.059 GHz and a Q factor reduction of 35 with respect to 1.042 GHz and 1000 with DI water, respectively.

Regarding EIS sensor experiments with NS solution, due to the higher conductivity with respect to DI water case, to model the EIS impedance data, a modification of the equivalent circuit was necessary, taking into account an R_s_ term related to the solution resistance and placed in series with the parallel CPE–R circuit.

Figure 5b shows trends of both the resonance frequency of CDSRR and resistance R extracted by fitting the EIS Nyquist plots. In addition, in these experiments, R was observed to increase when the amount of ethanol in the solution was increased, but presented much lower values than in DI water (Figure 3b). An almost linear trend of the resistance R was observed for EtOH concentrations lower than 50% and a sensitivity of 14.23 ± 0.28 Ω/% EtOH and a LOD of 0.6% EtOH were estimated by linear fitting the resistance curve in this range. These results further support the ability of the complex impedance fitting procedure to provide information on the ethanol content, also in the presence of contaminants which could indeed be identified through relevant changes in the equivalent circuit and its circuital parameters.

As far as CDSRR results in ethanol-saline solutions are concerned, these sensors showed a sensitivity of 0.177 ± 0.035 MHz/% EtOH and a LOD of 0.6% EtOH, demonstrating their efficacy in sensing ethanol in the presence of water-soluble contents, which had a recognizable effect on the Q factor.

## 4. Conclusions

In conclusion, we performed a comparison of miniaturized EIS and CDSRRs sensors performances for the detection of ethanol in water at very low percentages *v*/*v*. For each sensing architecture, two quantities were evaluated as readout parameters. In EIS sensors, we monitored variations in both the resistance R and the parameter Q_0_, related respectively to the electrical conductivity and dielectric properties of the investigated solution. On the other hand, perturbation of ring resonators gave access to working eigenfrequency shift and quality factor variation. Both the devices and related methods resulted in the efficient detection of low amounts of ethanol in water. The presence of salt in the solution influenced the sensor responses and performances but also resulted in recognizable effects on the output parameters, which provided information on the different environments. We concluded that EIS frequency analysis via an equivalent circuit proved to be effective to perform broadband evaluation of ethanol concentration in solution and it is convenient when low cost is the priority. On the other hand, the employment of resonant cavities allowed us to achieve a lower LOD and, in case of a known environment, can enable fast real-time single-frequency measurements.

## Figures and Tables

**Table 1:** Values of sensitivity and LOD of our EIS sensor and CDSRR, and comparison with other water/ethanol fluidic sensors in the literature.

Reference	Sensor	Sensitivity	LOD
[21]	MSR	*f*_res_ → 1.78 MHz/% EtOH	2.2% EtOH
[22]	SRR	*f*_res_ → 1.20 MHz/% EtOH	-
[25]	CSSR	*f*_res_ → 8.50 MHz/% EtOH	10% EtOH
[26]	OCSRR	*f*_res_ → 1.80 MHz/% EtOH	5% EtOH
[27]	λ/4 Res.	*f*_res_ → 2.98 MHz/% EtOH	5% EtOH
[28]	MTM	*f*_res_ → 3.20 MHz/% EtOH	0.005% EtOH
[49]	SRR	*f*_res_ → 0.89 MHz/% EtOH	10% EtOH
Our work	EIS	*Q*_0_ → 0.257 ± 0.008 pSHz^−n^/% EtOH	2.5% EtOH
		*R* → 5.74 ± 0.26 kΩ/% EtOH	0.7% EtOH
Our work	CDSRR	*f*_res_ → 0.329 ± 0.016 MHz/% EtOH	0.2% EtOH

## Data Availability

Data is contained within the article.
